# Globular-shaped variable lymphocyte receptors B antibody multimerized by a hydrophobic clustering in hagfish

**DOI:** 10.1038/s41598-018-29197-w

**Published:** 2018-07-17

**Authors:** Jaesung Kim, Se Pyeong Im, Jung Seok Lee, Jassy Mary S. Lazarte, Si Won Kim, Jae Wook Jung, Jong Yong Kim, Young Rim Kim, Sangmin Lee, Gwang Joong Kim, Hyun Suk Jung, Kyun Oh Lee, Alexandra Adams, Kim D. Thompson, Tae Sung Jung

**Affiliations:** 1 Laboratory of Aquatic Animal Diseases, Institute of Animal Medicine, College of Veterinary Medicine, Gyeongsang National University, 501 Jinju-daero, Jinju, 52828 South Korea; 2 Department of Biochemistry, College of Natural Sciences, Kangwon National University, 1 Kangwondaehak-gil, Chuncheon-si, Kangwon-do 24341 South Korea; 3 Division of Applied Life Science (BK21 + program), PMBBRC, Gyeongsang National University, Jinju, South Korea; 4 Institute of Aquaculture, University of Stirling, Stirling, FK9 4LA UK; 5 Moredun Research Institute, Pentlands Science Park, Bush Loan, Penicuik, EH26 0PZ UK

## Abstract

In hagfish and lampreys, two representative jawless vertebrates, the humoral immunity is directly mediated by variable lymphocyte receptors B (VLRBs). Both monomeric VLRBs are structurally and functionally similar, but their C-terminal tails differ: lamprey VLRB has a Cys-rich tail that forms disulfide-linked pentamers of dimers, contributing to its multivalency, whereas hagfish VLRB has a superhydrophobic tail of unknown structure. Here, we reveal that VLRBs obtained from hagfish plasma have a globular-shaped multimerized form (approximately 0.6 to 1.7 MDa) that is generated by hydrophobic clustering instead of covalent linkage. Electron microscopy (EM) and single-particle analysis showed that the multimerized VLRBs form globular-shaped clusters with an average diameter of 28.7 ± 2.2 nm. The presence of VLRBs in the complex was confirmed by immune-EM analysis using an anti-VLRB antibody. Furthermore, the hydrophobic hagfish C-terminus (HC) was capable of triggering multimerization and directing the cellular surface localization via a glycophosphatidylinositol linkage. Our results strongly suggest that the hagfish VLRB forms a previously unknown globular-shaped antibody. This novel identification of a structurally unusual VLRB complex may suggest that the adaptive immune system of hagfish differs from that of lamprey.

## Introduction

The adaptive immune system (AIS) evolved concomitantly with the emergence of vertebrate animals^1,2^. Unlike the jawed vertebrates, lampreys and hagfish, which are the only extant species representing the jawless vertebrates, appear to have independently evolved and uniquely employed leucine-rich repeat (LRR)-based variable lymphocyte receptors (VLRs) for their AISs against foreign antigens (Ags)^3,4^. Over the last decade, the VLRs have been consistently shown to play crucial roles in cellular (VLRA and VLRC) and humoral (VLRB) immunity^1–6^. During differentiation in response to particular Ags, germline *VLRs* are matured by combinatorial assembly with variable LRR-encoding cassettes in mutually exclusive populations of lymphocyte-like cells. Thereafter, somatic gene rearrangement of *LRR* offers the potential to generate a tremendous number of VLR variants to counteract numerous immunogens^3,4^. Although VLRBs share a common function with B-cell receptors, they are structurally more related to the LRR-family proteins, such as the toll-like receptors^3,4^. Each *VLRB* transcript encodes the following: a signal peptide (SP); the N-terminal LRR (LRRNT); LRR1; multiple variable LRR modules (LRRVs), including the end LRRV (LRRVe); a connecting peptide (CP); a C-terminal LRR (LRRCT); and a conserved C-terminus comprising a Thr/Pro-rich stalk region, a predicted glycophosphatidylinositol (GPI)-anchor site, and a hydrophobic tail^3,4^. Crystallographic characterizations of the interactions between VLRB and particulate Ags have predicted that the LRRVs and LRRCT are predominantly involved in specifically recognizing and binding to the corresponding Ags^7,8^. It has been suggested that the predicted GPI cleavage site enables the controlled release of VLRBs from lymphocytic membranes. Indeed, the cleavage of GPI-anchored lamprey VLRB expressed on the surface of transduced mouse thymoma cells was experimentally verified by treatment with bacterial GPI-specific phospholipase C^3^. Thus, VLRB appears to play a dual role as both a cell-surface receptor and as a humoral agglutinin in the AIS.

Since the middle of the 20^th^ century, results published by various researchers have shown that Ag-specific agglutinins and immunological memory can be produced by the AISs of lampreys and hagfish^9,10^. Such studies showed that fish immunized with various types of Ags could generate Ag-specific agglutinin molecules with molecular weights similar to that of an immunoglobulin (Ig) M antibody. This Ag-specific agglutination was explained by the discovery that lamprey VLRB is capable of multimerization^11^. Compared to IgG, multivalent lamprey VLRB showed a markedly higher binding avidity for its Ag; conversely, VLRB monomerized by deletion of its C-terminus completely lost its binding affinity^11^. To create the deca-valency (or octa-valency) of lamprey VLRB necessary for efficient binding, lamprey VLRBs form a pentamer (or tetramer) of dimeric subunits by disulfide cross-linkages that occur within their C-terminal Cys-rich tails. The lamprey VLRB complex reportedly has eight to ten identical Ag-binding sites and a structure resembling that of the IgM antibody^2,6,11^. In contrast, the native structure of hagfish VLRB has not yet been thoroughly documented. As the hagfish VLRB reportedly shows Ag-specific agglutination^9,10,12^, it should theoretically possess a certain domain for multimerization, or at least dimerization (as seen for IgG). However, no study to date has addressed how hagfish VLRB undergoes agglutination in response to specific Ags. Here, we for the first time describe a unique structure of multimerized VLRB antibodies in hagfish plasma and demonstrate that the multimerization of hagfish VLRB could be mediated by hydrophobic clustering directed by its C-terminal region.

## Results

### Multimerized hagfish VLRB by a hydrophobic clustering

**Figure 1:**
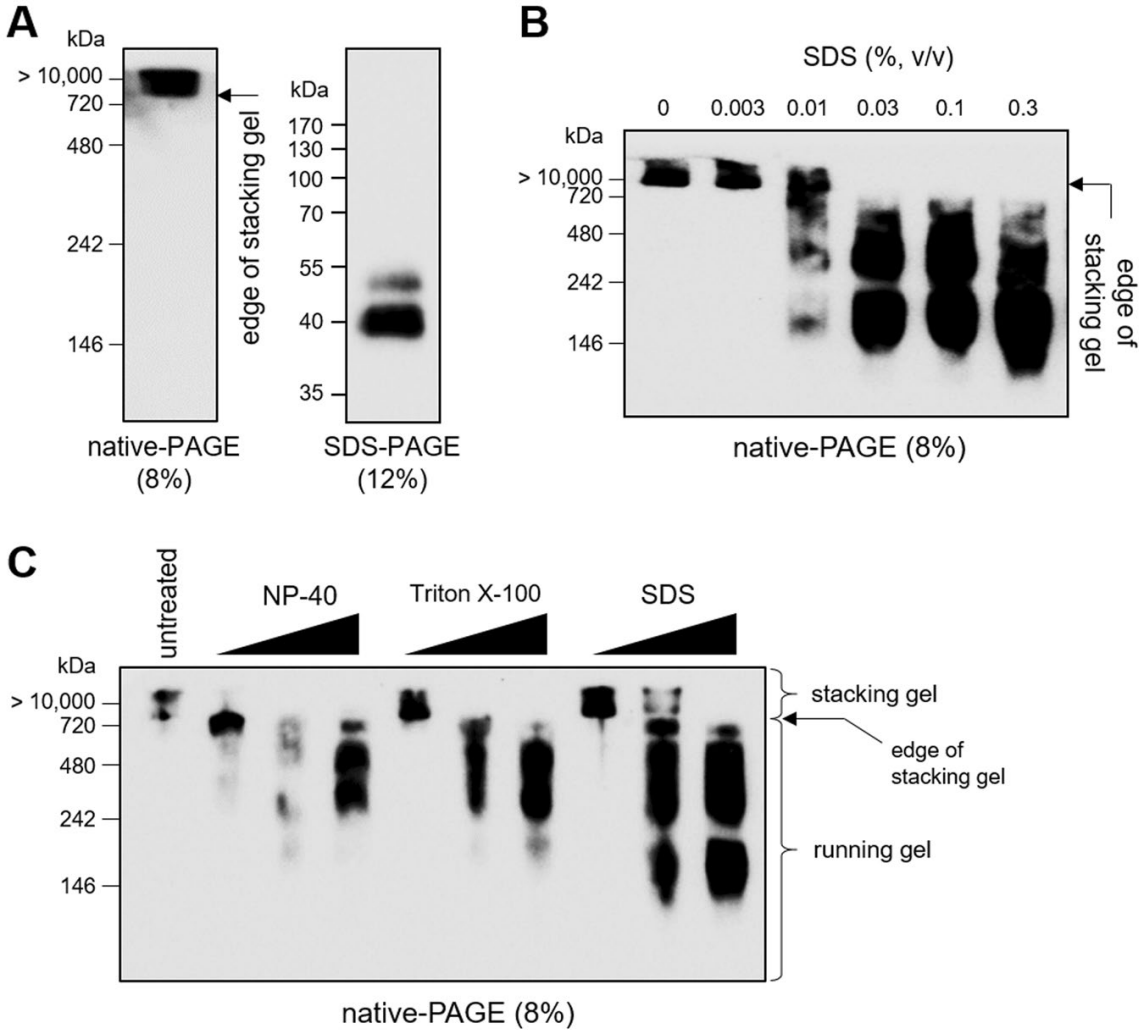
Characterization of multimerized VLRB by hydrophobic clustering in hagfish plasma. (**A**) Total hagfish plasma proteins (3.5 μg/lane) were separated by 8% native-PAGE (left) or 12% SDS-PAGE (right) under non-reducing conditions, followed by Western blot analysis with 11G5. (**B**) Western blot analysis of VLRB in total hagfish plasma proteins treated with SDS (0, 0.003, 0.01, 0.03, 0.1 or 0.3%, v/v) at room temperature for 10 min. (**C**) Hagfish plasma samples were incubated with NP-40 (0.01, 0.03, or 0.1%, v/v), Triton X-100 (0.005, 0.015, or 0.03%, v/v), or SDS (0.003, 0.01, or 0.03%, v/v) at room temperature for 10 min. The samples were resolved by native-PAGE (8%), and VLRBs were detected with 11G5 followed by goat anti-mouse IgG-HRP.

Although the lamprey and hagfish VLRBs share the same U-shaped structure and contain the same functional domains in the same order, from the signal peptide (SP) to the stalk region, their C-terminal tails differ (see Supplementary Fig. S1). The lamprey C-terminus (LC) possesses a Cys-rich region that can form disulfide-linked pentamers (or tetramers) of dimers, similar to the structure of the IgM antibody in mammals (Supplementary Fig. S1A)^2,11,13,14^. This multimerized organization of VLRB involves the formation of several disulfide bonds between each LC region; the resulting multivalency conferred by eight to ten identical Ag-binding subunits yields a remarkably high Ag-binding avidity^8,11,13,14^. In contrast, the hagfish C-terminus (HC) contains only one Cys and a cluster of hydrophobic amino acids (Supplementary Fig. S1B)^4,7^. No previous study had elucidated how the hagfish VLRB could also take on a multivalent, agglutination-forming structure^10,12^. To characterize a natural form of secreted hagfish VLRB, we separated hagfish plasma by native-PAGE or SDS-PAGE and subjected the resolved proteins to Western blotting with the mouse anti-VLRB monoclonal antibody, 11G5, which recognizes the invariant stalk region^15^. As shown in Fig. 1A (left), hagfish VLRB was detected exclusively at the edge of the native-PAGE stacking gel, and no migration was observed. When subjected to SDS-PAGE under non-reducing conditions, however, hagfish VLRB rapidly migrated as monomeric units of ~40 kDa (Fig. 1A, right), which was previously reported to be the approximate average molecular weight of an individual VLRB monomer^11,15^. The large VLRB complex was dramatically and dose-dependently disrupted by treatment with SDS (an ionic detergent) followed by native-PAGE (Fig. 1B). Similar, albeit weaker, dose-dependent disruptions of the VLRB complex were observed following treatment with non-ionic detergents, such as NP-40 or Triton X-100 (Fig. 1C). As these detergents are commonly used to denature proteins by disrupting non-covalent interaction and permeating into the hydrophobic core of native proteins^16,17^, these results suggest that circulating hagfish VLRB may naturally exist as multimeric complexes that lack covalent linkages, such as the disulfide bonds formed by the LC in lamprey.

### Globular-shaped VLRB complex particles in hagfish plasma

**Figure 2:**
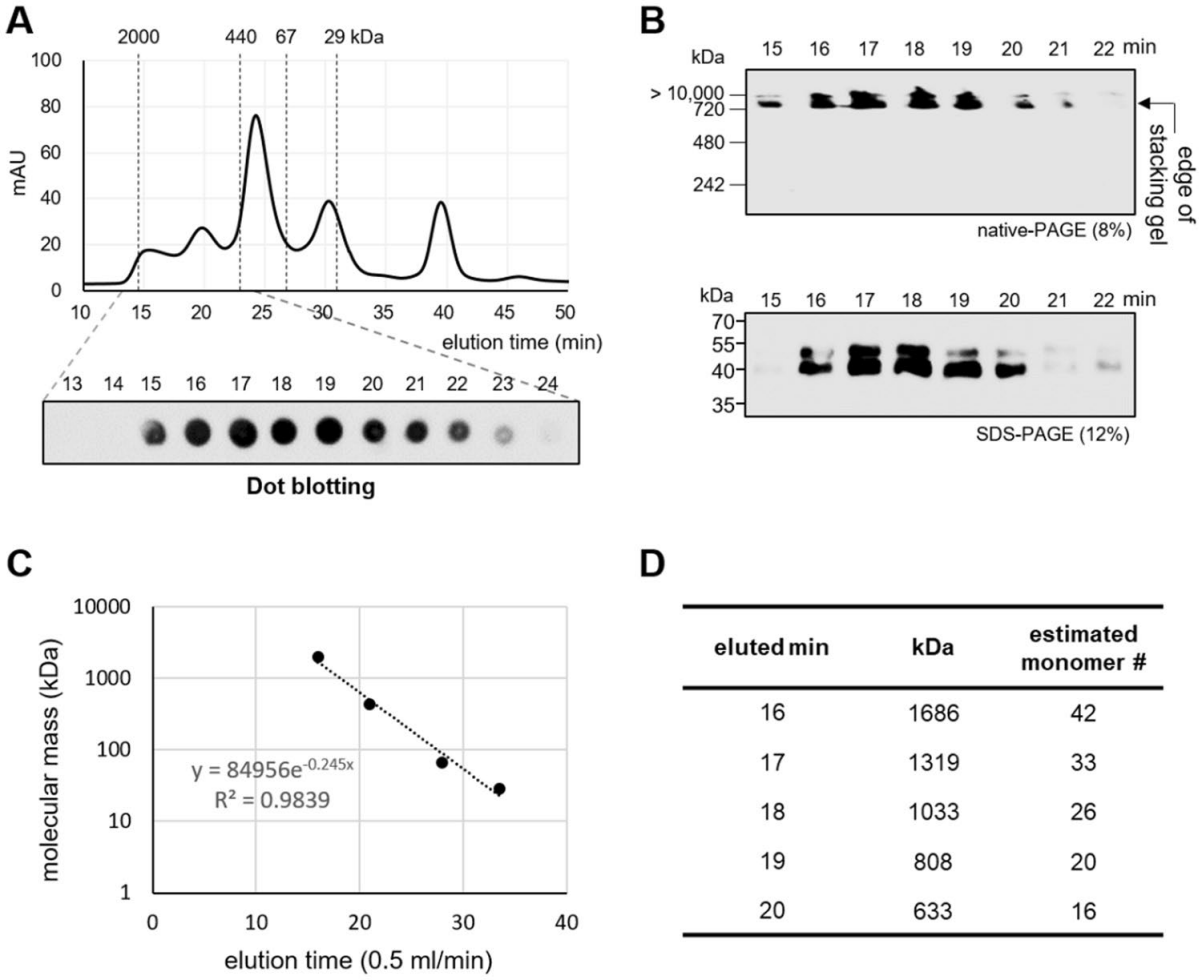
Size-dependent fractionation of total hagfish plasma proteins and the estimated molecular masses of the hagfish VLRBs fractionated by gel filtration. (**A**) Size-dependent fractionation of total hagfish plasma proteins by size-exclusion chromatography and dot blotting of the eluted samples. The eluted fractions (13~24 min) were analyzed by dot blotting with 11G5. (**B**) Western blotting of the fractionated samples (15~22 min). The fractionated plasma samples were run on native-PAGE or SDS-PAGE under non-reducing conditions, and subjected to Western blotting with 11G5. (**C**) Calibration of gel filtration standards. The calibration standards were blue dextran 2000 (2,000 kDa), ferritin (440 kDa), bovine serum albumin (BSA, 67 kDa), and carbonic anhydrase (29 kDa), all run at a flow rate of 0.5 ml/min. A linear regression of the elution profiles of the standards was plotted. (**D**) The elution time-based estimations of the molecular masses of the eluted complexes. Using the obtained equation (y = 84956e^−0.245x^), we estimated the molecular mass of each eluted complex based on the time (min). The fractions eluted from 16 to 20 min were found to harbor the major population of plasma VLRB, as shown in Fig. 2B. The number of the estimated VLRB monomers comprising each large VLRB complex was calculated using ~40 kDa as the average molecular mass of VLRB.

**Figure 3:**
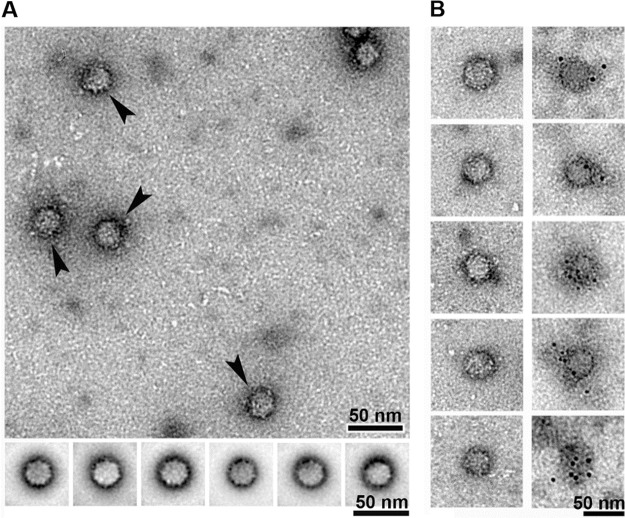
Electron microscopic analysis of the fractionated VLRB complex. (**A**) Shown are negatively stained fields and averaged images. (**B**) The negatively stained images of non-labeled particles (left vertical panel) and those labeled with immuno-gold using the anti-VLRB antibody (right vertical panel). Black arrowheads indicate the individual globular-shaped complex particles found in the field.

To obtain an apparent molecular mass for circulating VLRB, we fractionated whole plasma by size-exclusion chromatography (Fig. 2A). Time-dependent eluted fractions (1–48 min; 1 fraction per minute; flow rate, 0.5 ml/min) were screened by dot blotting (Fig. 2A) with 11G5, and the selected VLRB-positive fractions (15~22 min) were confirmed by Western blotting on native-PAGE or non-reducing SDS-PAGE (Fig. 2B). Consistent with the results obtained using whole plasma, the fractionated VLRBs failed to migrate on native-PAGE and migrated only as monomers on SDS-PAGE. A calibration plot performed using gel filtration standards estimated that the molecular mass of the main population (16–20 min fractions) of VLRB was distributed between approximately 600 kDa and 1.7 MDa (Fig. 2C,D). Given the average molecular mass of a VLRB monomer (40 kDa), this suggests that the hagfish VLRB circulating in blood might exist as large multimerized complexes of 16–42 monomers (Fig. 1A, right; Fig. 2D). We next used transmission electron microscopy (TEM) to examine the structures of the VLRBs in the 16–17 min fraction. The general appearances of highly populated particles in fields were visualized by negative staining (Fig. 3A), which revealed globular-shaped complexes with a diameter of 28.68 ± 2.16 nm (measured from 110 particles). Immune-electron microscopy using immuno-gold labeling with 11G5 revealed enrichment of gold-conjugated anti-VLRB antibodies on the surfaces of these complexes, confirming the presence of VLRBs. (Fig. 3B). Together, these results suggest that an intermolecular interaction among hagfish VLRBs forms a globular-shaped multimerized complex in plasma.

### Secretion of multimerized recombinant VLRBs

**Figure 4:**
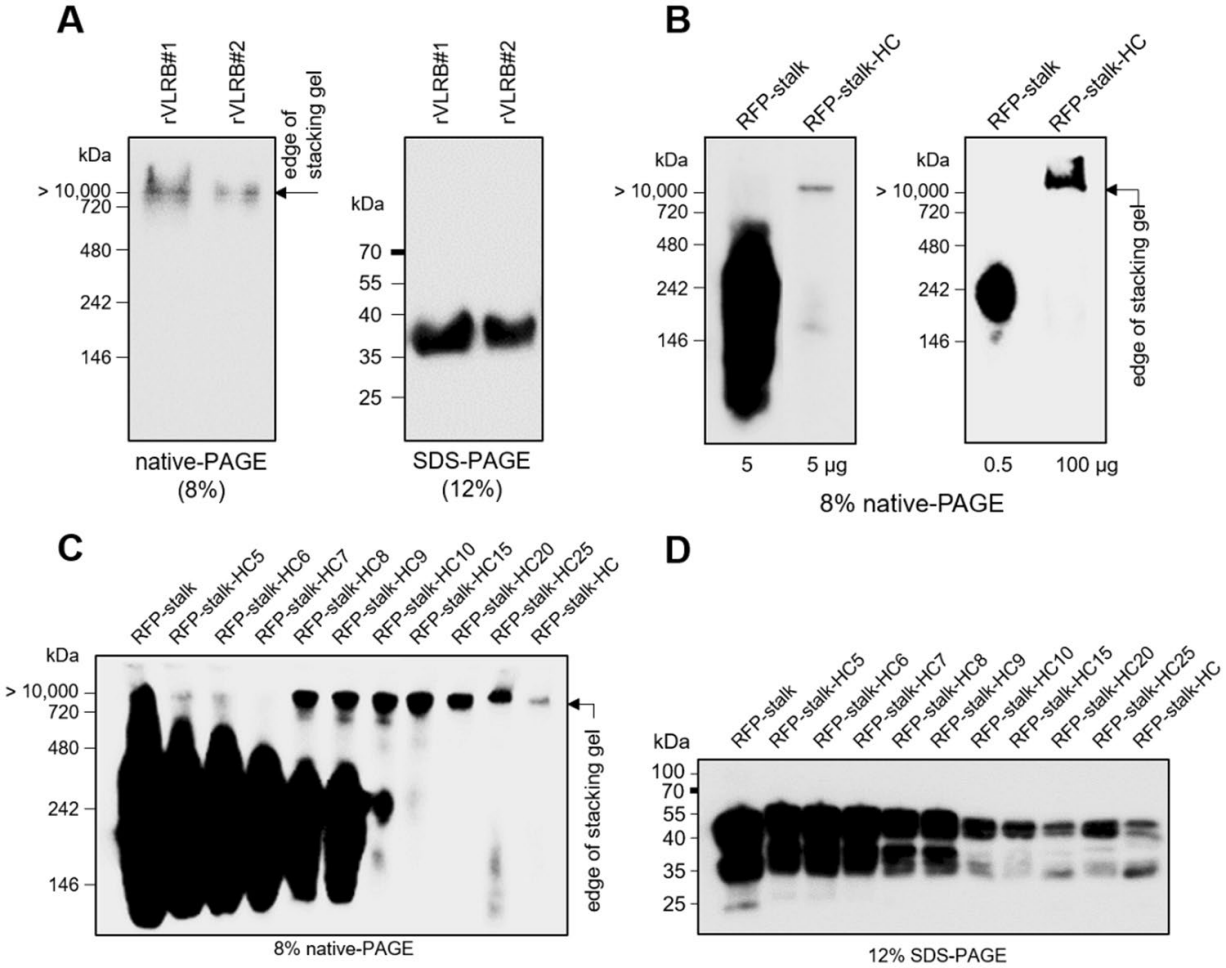
Multimerization triggered by the HC. (**A**) Western blotting of the secreted recombinant VLRBs. The secretion of the randomly selected rVLRBs (rVLRB#1, rVLRB#2) was visualized by Western blotting with 11G5 after running on nave-PAGE (8%) or SDS-PAGE (12%). (**B**) Western blotting of RFP-stalk or RFP-stalk-HC. Each supernatant (5 and 0.5 μg for RFP-stalk; 5 and 100 μg for RFP-stalk-HC) was separated by 8% native-PAGE under non-reducing condition, followed by Western blot analysis with 11G5. The original full-length blot is presented in Supplementary Fig. S2. (**C**) Minimum requirement of the HC8 for multimerization of RFP-stalk. Supernatants from 293-F cells transfected with the plasmids expressing RFP-stalk with or without various versions of the HC (HC5, HC6, HC7, HC8, HC9, HC10, HC15, HC20, HC25, or HC; see Supplementary Table S1) were run on 8% native-PAGE, and subjected to Western blot analysis with 11G5. (**D**) Reduced expression levels depending on the sizes of the conjugated HC. Each supernatant was separated by 12% SDS-PAGE under non-reducing conditions followed by Western blotting with 11G5.

To test whether hagfish recombinant VLRBs (rVLRBs) could spontaneously form multimeric complexes, 293-F cells were transfected with vectors encoding two rVLRB proteins (rVLRB#1 or rVLRB#2) and the secreted proteins were assessed by Western blot analysis (Fig. 4A). Consistent with the results obtained from hagfish serum, non-migratory multimerized rVLRB complexes were exclusively observed following native-PAGE (Fig. 4A, left), while the monomers were exclusively observed on non-reducing SDS-PAGE (Fig. 4A, right). However, the secreted rVLRBs were not detectable under our initial conditions (data not shown); we were only able to visualize them after they were concentrated 20-fold by freeze drying. Their expression was dramatically increased by deletion of the HC, suggesting that the strong hydrophobic region in the HC decreased the expression efficiency of the rVLRBs. The monomerized rVLRBs could be observed on SDS-PAGE after 20-fold concentration, but the multimerized rVLRBs were barely visible on native-PAGE even after concentration. Treatment of the multimerized rVLRBs with a very low concentration of SDS (0.003%, v/v) allowed them to produce weak signals on Western blots, presumably because the detergent somewhat weakened the compact structure of the globular rVLRB complexes. We speculate that the HC-adjoining stalk region, which is specifically recognized by 11G5, is shielded by multimerization, which exposes the Ag-binding regions (LRRNT to LRRCT) while sequestering the stalk to the hydrophobic core of the globular rVLRB structure. This indicates that the multimerized rVLRB complexes were not generated by covalent bonds between individual VLRB monomers, but instead were clustered via the hydrophobic region of the HC. The latter conclusion is supported by our observation that, again similar to the serum complexes, the recombinant complexes could be disrupted by detergent treatment, as observed in Fig. 1B,C.

### Triggering multimerization by the HC

To verify that the HC plays a key role in multimer formation, we examined the complexation of secreted RFP reporter proteins conjugated only with the stalk (RFP-stalk) or with stalk-HC (RFP-stalk-HC) (Fig. 4B, Supplementary Fig. 2). Similar to the results obtained for rVLRBs, RFP-stalk-HC was successfully secreted, albeit at a low level, as a multimerized form, whereas RFP-stalk (analogous to the HC-truncated rVLRB) showed a higher-level expression of solely monomeric units. To evaluate the minimal region required for multimerization, we serially deleted portions of the HC-encoding sequence from the vector encoding RFP-stalk-HC (see Supplementary Table S1). Although the expression and secretion of the RFP-stalk-HC variants decreased with the length of the HC on native-PAGE (Fig. 4C) or on non-reducing SDS-PAGE (Fig. 4D), their multimerization relied totally upon the length of the conjugated HC; it was first seen with HC8 (YFPSYIFL) and fully enabled by HC15 (YFPSYIFLHLVHGLA). We do not yet know how many monomeric units participated in the multimerization of each complex (RFP-HC8 to RFP-HC), but these results clearly show that the HC enables RFP-stalk to undergo multimerization via the previously reported phenomenon of hydrophobic clustering^18,19^.

### Cellular surface localization via GPI linkage of the HC

**Figure 5:**
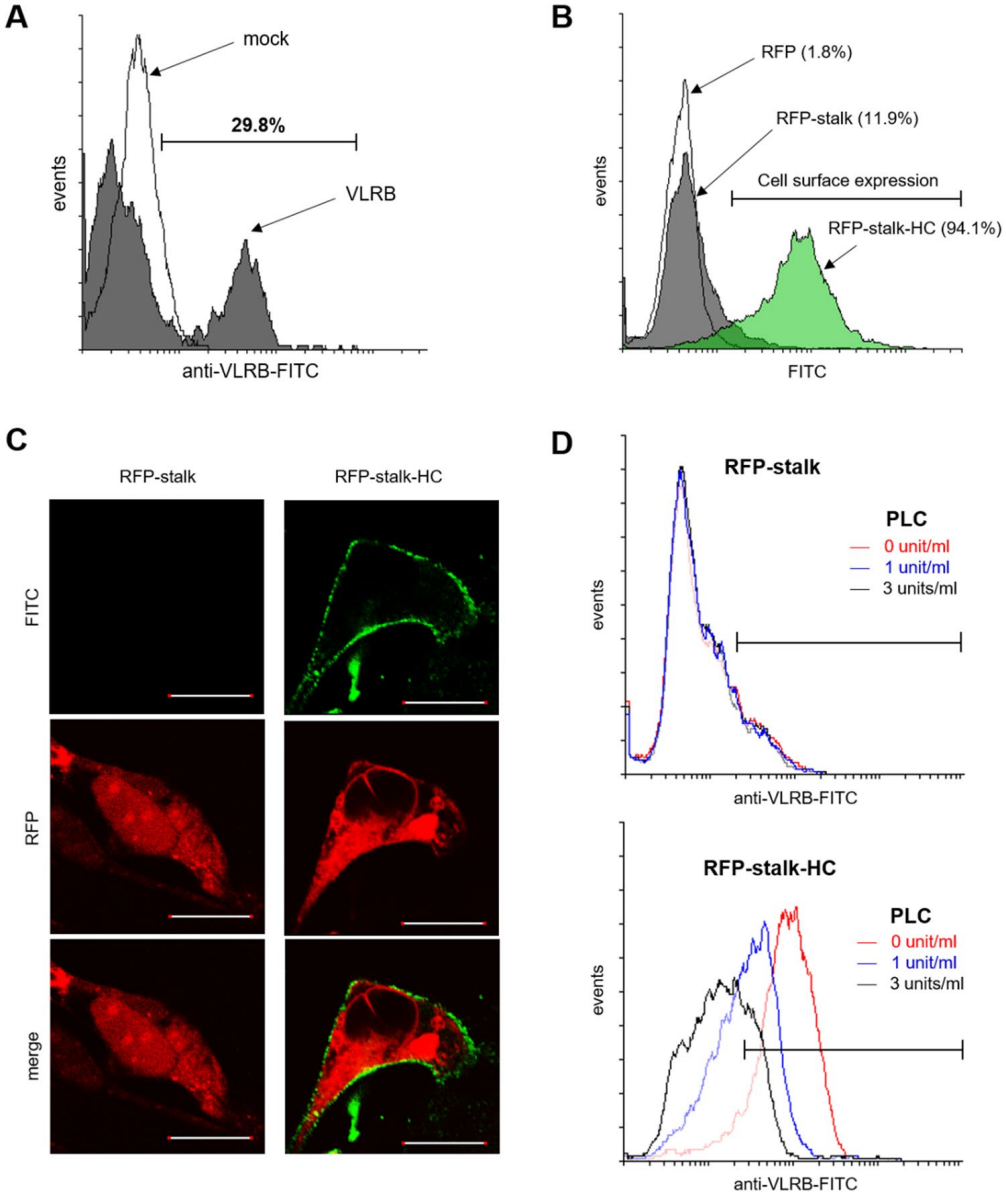
Requirement of the HC for cell surface via GPI anchoring. (**A**) Cellular surface localization of VLRB on the lymphocyte-like cells of hagfish by flow cytomery. The purified lymphocyte-like cells were incubated with 11G5, followed by incubation with goat anti-mouse IgG-FITC after each step washing. The cells incubated only with the secondary antibody (negative control: mock, black line) had a positive population of 1.7%, whereas approximately 29.8% of the experimental cell population (gray-shaded) was positive for surface-displayed VLRB. (**B**) Cell surface expression analyzed by by flow cytometry. Cell surface localization of the 293-F cells was assessed by flow cytometry and detected by 11G5 followed by goat anti-mouse IgG-FITC. Gating revealed that 1.8%, 11.9%, and 94.1% of the cells transfected with pKIN/RFP, pKIN/RFP-stalk, and pKIN/RFP-stalk-HC, respectively. These values indicate the populations of cells showing surface localization. (**C**) Confocal images of 293-F cells expressing RFP-stalk or RFP-stalk-HC. At 48 hrs after transfection, the cells were incubated with 11G5 followed by goat anti-mouse IgG-FITC. The FITC-stained cells were visualized by confocal imaging. (**D**) Cell surface localization via GPI anchoring. Transfected 293-F cells expressing RFP-stalk (upper) or RFP-stalk-HC (lower) were treated with 0, 1 or 3 units/ml of bacterial GPI-specific phospholipase C, the surface-localized stalk regions were stained with 11G5 followed by anti-mouse IgG-FITC, and the cells were subjected to FACS analysis.

As lamprey VLRB can be found both in plasma and displayed on the surface of VLRB + lymphocytes (15–35%) in blood^20^, we examined whether hagfish VLRB was also found on the cell surfaces of VLRB + lymphocytes. Similar to the results in lamprey, we found that about 30% of the lymphocyte-like cells in hagfish exhibited surface expression of VLRB (Fig. 5A). To determine whether the HC could trigger this cell surface localization, we used flow cytometry to analyze the surface-displaying populations of 293-F cells expressing RFP-stalk or RFP-stalk-HC (Fig. 5B). Compared with the populations of cells expressing RFP (1.8%) or RFP-stalk (11.9%), almost all of the relevant cells exhibited surface expression of RFP-stalk-HC (94.1%). The surface localization of RFP-stalk-HC was confirmed by confocal imaging analysis (Fig. 5C). To verify that the displayed RFP-stalk-HC was linked via GPI anchoring, cells expressing RFP-stalk or RFP-stalk-HC were treated with or without GPI-specific phospholipase C (PLC) and analyzed by immunofluorescence (Fig. 5D). Indeed, PLC treatment dose-dependently induced the release of RFP-stalk-HC, but not RFP-stalk, from the cell surface. Taken together, our results show that RFP-stalk-HC can be displayed on the surface of the transfected cells and can be secreted as a multimer, whereas RFP-stalk can only be secreted as a monomer. We thus conclude that hagfish VLRBs do not solely exist in plasma as humoral agglutinins, but can also be localized on the surface of lymphocyte-like cells as cellular receptors. Moreover, the HC can drive both the cell surface localization of hagfish VLRB via GPI anchoring and the globular multimerization of hagfish VLRB via hydrophobic clustering.

## Discussion

Hydrophobic interaction has long been considered one of the most important binding forces for protein folding, adhesion, aggregation, and self-assembly. Unlike fibrous and membrane proteins, globular (or spherical) protein complexes are generally soluble in water, forming colloids wherein globular assembly is driven by the hydrophilic and hydrophobic properties of the protein, in a manner similar to micelle formation^18,19^. Our results demonstrate that individual VLRB monomers could be tightly packed together by self-assembly via hydrophobic clustering of the HC. We believe that the stalks linked to the hydrophobic regions of the hagfish HC are buried inside the complex, while the Ag-binding regions (between LRRNT and LRRCT) are exposed on the outer surface, allowing dipole-dipole interactions to ensure their solubility and outward Ag-binding space. In our previous study, this multivalency enabled us to use ELISA to show time- and dose-dependent Ag-specific VLRB responses in the plasma of immunized hagfish^15^. However, our efforts to generate an Ag-specific monoclonal rVLRB antibody using different mammalian cells (293-F, 293 T, CHO, and COS-7) have been unsuccessful even when we immunize the hagfish with variable Ags, such as viral hemorrhagic septicemia virus (VHSV), avian influenza virus hemagglutinin (H9N2-HA1), etc. (data not shown). Whereas VLRBs are abundant in hagfish plasma samples, the rVLRB multimers artificially secreted from mammalian cells were expressed at too low a level to be detected by ELISA and Western blotting as well. Moreover, the burying of the stalk region, which is a unique characteristic of the globular VLRB formation, might decrease the ability of 11G5 to reach its recognition site. Hence, epitope tagging of the rVLRB N-terminus or the generation of LRRNT-specific mouse monoclonal Abs might facilitate further efforts to discover Ag-specific rVLRB clones.

**Figure 6:**
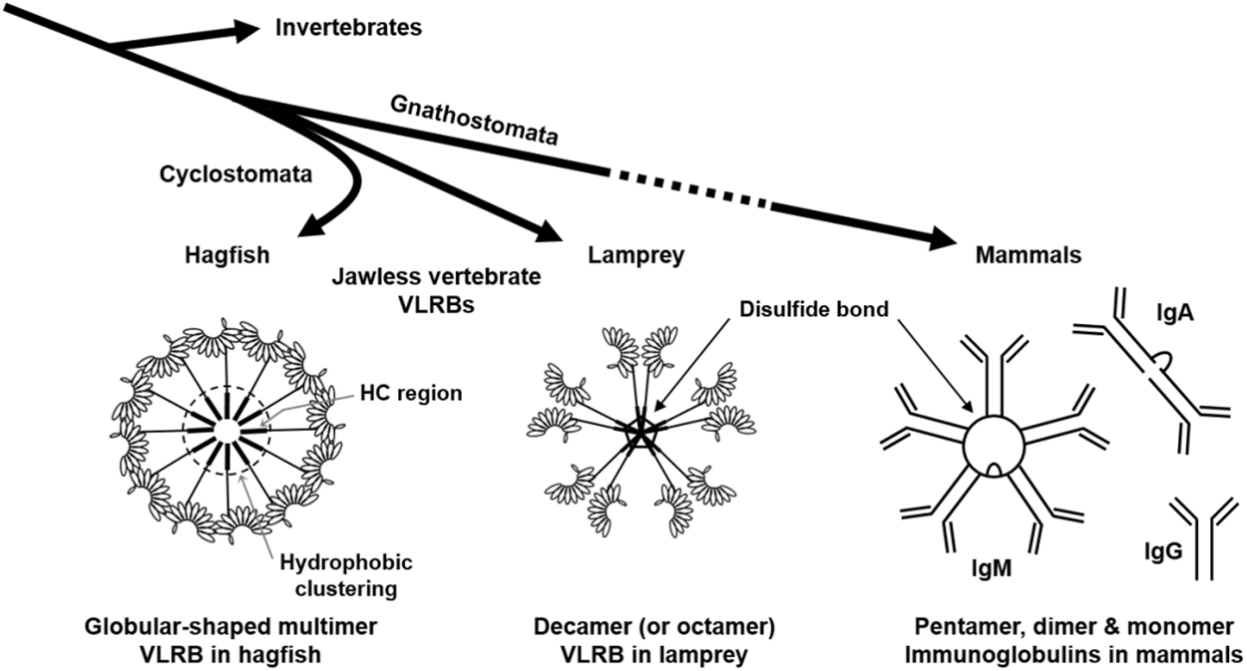
Schematic representation of secreted VLRB antibodies in jawless vertebrates (hagfish and lamprey) and immunoglobulin antibodies in mammals.

Morphologists and molecular biologists have long disagreed on the relationships among lamprey, hagfish, and gnathostomes^21,22^. Even phylogenetic analyses using combined phenotypic-molecular characteristic data sets have failed to fully clarify if lampreys are more closely related to gnathostomes than hagfish, or if lampreys and hagfish are sister groups^23^. This discrepancy means that there is still controversy at the very base of the vertebrate tree^21^. However, from a morphological perspective, if lamprey diverged from hagfish, then the globular-shaped hagfish VLRB may represent a primordial antibody structure in jawless and jawed vertebrates. We also do not yet know if the globular-shaped hagfish VLRB is more evolved for adaptive immunity than the pentagonal-shaped lamprey VLRB. We know that the pentagonal-shaped lamprey VLRB bears a closer resemblance to mammalian IgM compared to globular-shaped hagfish VLRB. Conversely, hagfish VLRBs have higher multivalency due to the presence of more Ag-binding sites, and thus could have a higher binding avidity for Ags. Another speculation is that the AIS mediated by the hagfish antibody might exist alongside one or more other immune mechanisms (i.e., a complement system), further distinguishing hagfish from lamprey and jawed vertebrates. Our present results do not allow us to say that the globular-shaped hagfish VLRB is a more or less efficient structure for Ag-binding than the pentamer-shaped lamprey VLRB or even immunoglobulins in mammals (Fig. 6). They do, however, suggest the novel notion that there may exist an undiscovered acquired immune response mechanism in hagfish, based on the globular-shaped VLRB antibody complex.

## Methods

### Animals and cells

Inshore hagfish (*Eptatretus burgeri*) of 20~30 cm in size were freshly captured by commercial fishermen (Bogyeong Hagfish Serves, South Korea) and maintained in aquariums at 14~15 °C. The fish were anesthetized by immersion with ethyl 3-aminobenzoate methanesulfonic acid (0.1 g/L; Sigma), and whole blood was collected and diluted with an equal volume of 0.67 × PBS in 10 mM EDTA, as previously described^15^. All experiments were reviewed and approved by the Institutional Animal Care and Use Committee at Gyeongsang National University. The plasma was harvested by centrifugation for 10 min at 500 × g, and the proteins were quantified using a Pierce BCA Protein Assay Kit (#23225, Thermo Scientific). Human embryonic kidney 293-F cells were purchased from Gibco (#11625-019, Life Technologies) and maintained in high-glucose Dulbecco’s Modified Eagle’s Medium (DMEM) containing 10% fetal bovine serum (FBS) in a 37 °C incubator with 5% CO_2_.

### Western blot analysis of hagfish plasma proteins

Hagfish plasma samples (3.5 μg/lane) were separated by 8% native-PAGE or 12% SDS-PAGE under non-reducing conditions, and transferred to methanol-activated PVDF membranes. The membranes were blocked with 5% skim milk in PBST (0.1% Tween 20 in PBS), and then incubated for 1 h with mouse anti-VLRB IgG1 (11G5), which was previously reported to recognize the invariant stalk region of hagfish VLRB^15^. The blots were then incubated with horseradish peroxidase (HRP)-conjugated goat anti-mouse IgG (Thermo Scientific), and the results were visualized using a SuperSignal West Pico Chemiluminescent Substrate kit (#34080, Thermo Scientific). After each incubation, the membranes were washed three times with PBST, for 30 min each time. To test the ability of a detergent to disrupt the VLRB multimers, the plasma samples were treated with different concentrations of SDS (sodium dodecyl sulfate: 0, 0.003, 0.01, 0.03, 0.1 or 0.3%, v/v) at room temperature for 10 min. In an attempt to visualize the multimerized rVLRBs following native-PAGE, the protein was concentrated 20-fold by freeze drying and treated with SDS (0.003%, v/v) at room temperature for 10 min prior to electrophoresis.

### Size exclusion chromatography and immunoblotting

One hundred micrograms of hagfish plasma were diluted in 600 μl of PBS, filtered with a 0.2-μm membrane, and then run on a Superdex 200 10/300 GL column (GE Healthcare). Fractions (500 μl) were harvested every minute from 1 min to 48 min (1 fraction per minute; flow rate, 0.5 ml/min), and dot blotting was used to select the VLRB-positive fractions. Since the eluted fractions showed very weak signals, all fractions were concentrated 20-fold by freeze drying before dot blotting. The concentrated fractions (1 min to 48 min) were mixed with 5 × SDS-loading buffer, boiled, and dotted on a 100% methanol-activated PVDF membrane. The membrane was blocked and incubated with 11G5 followed by an HRP-conjugated secondary Ab. The size-exclusion chromatography and dot blotting experiments were independently repeated four times using different plasma samples. The gel filtration standards used for calibration included Blue dextran 2000 (2,000 kDa), ferritin (440 kDa), bovine serum albumin (BSA, 67 kDa), and carbonic anhydrase (29 kDa). Linear regression performed using the individual elution times of the standards (214.7 min, 22.9 min, 26.4 kDa, and 30.5 min, respectively) yielded the equation y = 84956e^−0.245x^. The major population of VLRB was distributed in the 16–20 min fractions. The selected fractions were analyzed by dot blotting with 11G5 and by native-PAGE (8%) or SDS-PAGE (12%) under non-reducing conditions followed by Western blot analysis with 11G5.

### Transmission electron microscopy and single-particle image processing

The VLRBs of the 16–17 min fraction were diluted 100-fold with 1 × PBS to give a final concentration of 50 nM. Five microliters of the sample were applied to carbon-coated grids that had been glow-discharged (Harrick Plasma) for 3 min in air, and the grids were immediately subjected to negative staining with 1% uranyl acetate^24,25^. For immuno-gold labeling electron microscopy, the VLRBs (50 nM) were mixed with purified 11G5 for 1 h at a ratio of 1:2, and then treated with a secondary antibody that had been conjugated with 6-nm gold particles (Abcam). The final mixture was incubated overnight on ice and then negatively stained as described above. The samples were visualized using a Tecnai T10 transmission electron microscope (FEI) operated at 100 kV. Images were recorded with a 2 K × 2 K, Gatan US1000 CCD camera at a magnification of 34,000 (0.32 nm/pixel). Single-particle image processing was carried out using SPIDER (Health Research). The particles in the micrographs were picked manually from 180 × 180-pixel boxes, and the picked particle images were masked and normalized to eliminate any variation in image density. A total of 284 particles corresponding to negatively stained VLRBs were processed for class averaging^26^.

### Construction of plasmids

The pTracer-EF/V5-His mammalian expression vector (#V887-20, Invitrogen Life Technologies) was modified to introduce a murine Ig κ-chain leader sequence (Igκ) and two Sfi I sites. The DNA fragment flanked by Kpn I (bold) and two Sfi I (underlined)/Not I (italicized) sites was PCR amplified (forward primer: 5′-CA**GGTACC**ATGGAGACAGACACACTCCTG-3′; reverse primer: 5′-T*GCGGCCGC*GGGCCCCAGAGGCCTTTTTTGGCCCCGGTGGCCGAGTCACCAGTGGAACCTGGAAC-3′) from the template, pSecTac2A (#V900-20, Invitrogen Life Technologies). The PCR product was digested with Kpn I/Not I and ligated into the Kpn I/Not I sites of pTracer-EF/V5-His to generate pKINGeo. For more efficient cloning and to exclude self-ligation, a PCR product harboring the chloramphenicol resistance/ccdB gene flanked with Sfi I sites was amplified from pEF-DEST51 (#12285-011, Invitrogen Life Technologies) (forward primer: 5′-AGGCCACCGGGGCCAAAAAAGGCTTATGGAGAAAAAAATC-3′; reverse primer: 5′-AGGCCCCAGAGGCCTTATATTCCCCAGAACATCAG-3′). The obtained product was digested with Sfi I and inserted into the two Sfi I sites of pKINGeo to construct pKINGeo/ccdB. All generated constructs were verified by DNA sequencing analysis. Two selected VLRB cDNAs (from LRRNT to the stop codon of C-terminal region of the hydrophobic tail) purified from leukocytes of hagfish blood were cloned into the Sfi I sites of pKINGeo/ccdB using appropriate primers (forward: 5′-AGGCCACCGGGGCCTGTCCTTCACGGTGTTCCTG-3′; reverse: 5′-TGGCCCCAGAGGCCTCAGAGCAGCTGCGAGGCGT-3′) to generate pKIN/rVLRB#1 and pKIN/rVLRB#2. The inserted VLRBs (rVLRB#1, rVLRB#2) were verified by DNA sequencing. Both rVLRB#1 and rVLRB#2 included the LRRNT, one LRRV module, one LRRVe module, CP, LRRCT, the stalk, and the hydrophobic tail. To construct plasmids expressing RFP (mCherry) fused with the stalk-HC region of hagfish VLRB, the GFP-encoding fragment was removed from pKINGeo by Nde I digestion, and pKINGeo was self-ligated. Primers A, B, C, and D (A, forward: 5′-CGGCCACCGGGGCCATGGTGAGCAAGGGCGAGGA-3′; B, reverse: 5′-TAGTCGTAGTCTTGTACAGCTCGTCCATGC-3′; C, forward: 5′-GCTGTACAAGACTACGACTACCACTACCAC-3′; D, reverse: 5′-TGGCCCCAGAGGCCTCAGAGCAGCTGCGAGGCGT-3′) were used to amplify and fuse the fragments encoding RFP and HC. Briefly, primers A/B and C/D were used to separately amplify RFP and pKIN/rVLRB#1, respectively, and then the obtained products were mixed and amplified using primers A/D. The final PCR product (960 bp) was digested with Sfi I and inserted into the Sfi I sites of pKINGeo/ccdB to generate pKIN/RFP-stalk-HC. To construct pRFP or pRFP-stalk, the PCR products generated with primer A plus the reverse RFP primer (5′-TGGCCCCAGAGGCCTCACTTGTACAGCTCGTCCATGC-3′) or the reverse stalk primer (5′-AGGCCCCAGAGGCCTCACGCGTTCATGACACGGCCGA-3′) were digested with Sfi I and inserted into pKINGeo/ccdB to construct pKIN/RFP and pKIN/RFP-stalk, respectively. To generate serially truncated HC variants conjugated with RFP, the desired fragments were amplified using the forward RFP primer and various truncation-specific primers (see Supplementary Table S1). The PCR products were digested with Sfi I and cloned into the Sfi I sites of pKINGeo/ccdB.

### Transfection

The constructed plasmids were purified using DNA Spin miniprep kits (#17098, iNtRON Biotechnology) and quantified using a NanoDrop spectrophotometer. For transfection, 293-F cells were seeded to 24-well plates, grown to 90% confluent, and transfected with the plasmids using Lipofectamine2000 (#11668-019, Invitrogen) according to the manufacturer’s protocol. After 4 h, the transfectants were transferred to expression medium (#12338-018, Gibco Life Technologies). After 48 or 72 h, each supernatant was harvested and centrifuged for removal of cells and debris. When there were issues with very low-level expression (i.e., rVLRBs harboring the hydrophobic region), the supernatant was concentrated 20-fold by freeze drying and resuspended with RNase/DNase free distilled water.

### Western blotting of the secreted recombinant VLRBs (rVLRB#1, rVLRB#2)

From a VLRB cDNA library generated from unimmunized hagfish, we randomly selected 20 VLRB clones and tested their expression levels. We selected two clones (rVLRB#1, rVLRB#2) that showed relatively high expression levels and encoded LRRVs (including LRRVe) that we had previously identified as being among the most frequently observed LRRV modules. The secreted rVLRBs were harvested at 72 h post-transfection, concentrated 20-fold by freeze drying, treated with SDS (0.003%, v/v), separated by native-PAGE (8%) or SDS-PAGE (12%), and detected by Western blotting with 11G5.

### Western blot of RFP-stalk or RFP-stalk-HC

Two days after transfection of 293-F cells with pKIN/RFP-stalk or pKIN/RFP-stalk-HC, 5 μg of each supernatant was separated by 8% native-PAGE under non-reducing condition, followed by Western blot analysis with 11G5. Since the expression level of the hydrophobic HC-conjugated RFP-stalk (RFP-stalk-HC) was extremely low compared to that of RFP-stalk, we compared bands in diluted RFP-stalk supernatant (0.5 μg/well) versus freeze dry-concentrated RFP-stalk-HC (100 μg/well).

### Flow cytometry

293-F cells were grown to 90% confluent on 6-well plates, transfected with pKIN/RFP-stalk or pKIN/RFP-stalk-HC using Lipofectamine2000, incubated for 4 h, and then transferred to DMEM containing 10% FBS. After 48 h, the transfected cells were harvested, blocked with 0.1% BSA in PBS for 30 min at 4 °C, incubated with 11G5 for 1 h at 4 °C, washed three times with PBS, and then incubated with anti-mouse IgG-FITC for 30 min at 4 °C under foil. The cells were then washed, resuspended with PBS, and analyzed using a FACSCalibur™ (BD Biosciences). The population of the transfected cells expressing RFP was gated and regarded as 100%.

### Confocal microscopy

293-F cells were grown to 70% confluent on an 8-chamber slide incubated for 24 h, and transfected with pKIN/RFP-stalk or pKIN/RFP-stalk-HC using Lipofectamine2000. After 4 h, the transfectants were transferred to expression medium. After 48 h, the transfected cells were fixed with 4% paraformaldehyde in PBS, blocked with 0.1% BSA in PBS for 30 min, incubated with 11G5 for 1 h, washed three times with PBS, and incubated with anti-mouse IgG-FITC for 30 min. The slides were mounted with Vectashield (H-1000, Vector Labs), and images were viewed under a confocal microscope (Zeiss Axiovert).

### Bacterial GPI-specific phospholipase C (PLC) treatment

293-F cells were transfected with plasmids encoding RFP-stalk or RFP-stalk-HC for 2 days, and then the cells were harvested and treated with 0, 1, or 3 units/ml of PLC (Sigma, P5542-5 UN) for 45 min at 30 °C^3^. The PLC-treated cells were incubated with 11G5 followed by anti-mouse IgG-FITC, and then subjected to flow cytometry. The population of the RFP-expressing cells were gated and the FITC-positive population was evaluated.

### Data availability

All data generated or analyzed during this study are included in this published article and its Supplementary Information files.

## Electronic supplementary material

Supplementary Information
