# Supplementary material for: Globular-shaped variable lymphocyte receptors B antibody multimerized by a hydrophobic clustering in hagfish
Source: Sci Rep. 2018 Jul 17;8:10801. doi: 10.1038/s41598-018-29197-w (PMC6050320; doi:10.1038/s41598-018-29197-w)
Supplement: Supplementary file 1 — Supplementary Information [file 41598_2018_29197_MOESM1_ESM.docx]

**Globular-shaped variable lymphocyte receptors B antibody multimerized by a hydrophobic clustering in hagfish**

Jaesung Kim^1,+^, Se Pyeong Im^1,+^, Jung Seok Lee^1^, Jassy Mary S. Lazarte^1^, Si Won Kim^1^, Jae Wook Jung^1^, Jong Yong Kim^1^, Young Rim Kim^1^, Sangmin Lee^2^, Gwang Joong Kim^2^, Hyun Suk Jung^2^, Kyun Oh Lee^3^, Alexandra Adams^4^, Kim D. Thompson^5^ and Tae Sung Jung^1,*^

^1^Laboratory of Aquatic Animal Diseases, Institute of Animal Medicine, College of Veterinary Medicine, Gyeongsang National University, 501 Jinju-daero, Jinju, 52828, South Korea.

^2^Department of Biochemistry, College of Natural Sciences, Kangwon National University, 1 Kangwondaehak-gil, Chuncheon-si, Kangwon-do, 24341, South Korea

^3^Division of Applied Life Science (BK21 + program), PMBBRC, Gyeongsang National University, 501 Jinju-daero, Jinju, 52828, South Korea.

^4^Institute of Aquaculture, University of Stirling, Stirling FK9 4LA, UK

^5^Moredun Research Institute, Pentlands Science Park, Bush Loan, Penicuik EH26 0PZ, UK

^*^Address correspondence to Tae Sung Jung, jungts@gnu.ac.kr

^+^these authors contributed equally to this work.

**SUPPLEMENTARY TABLES**

**Table S1. Peptide sequences of the serially deleted HC variants that were fused onto the RFP-stalk, and the reverse primers used for their for PCR amplification.** Sfi I site (underlined)-flanked PCR products were generated using the forward primer for the N-terminus of RFP (5’-CGGCCACCGGGGCCATGGTGAGCAAGGGCGAGGA-3’) and the indicated reverse primers. The generated products were digested with Sfi I and cloned into the Sfi I sites of pKINGeo.


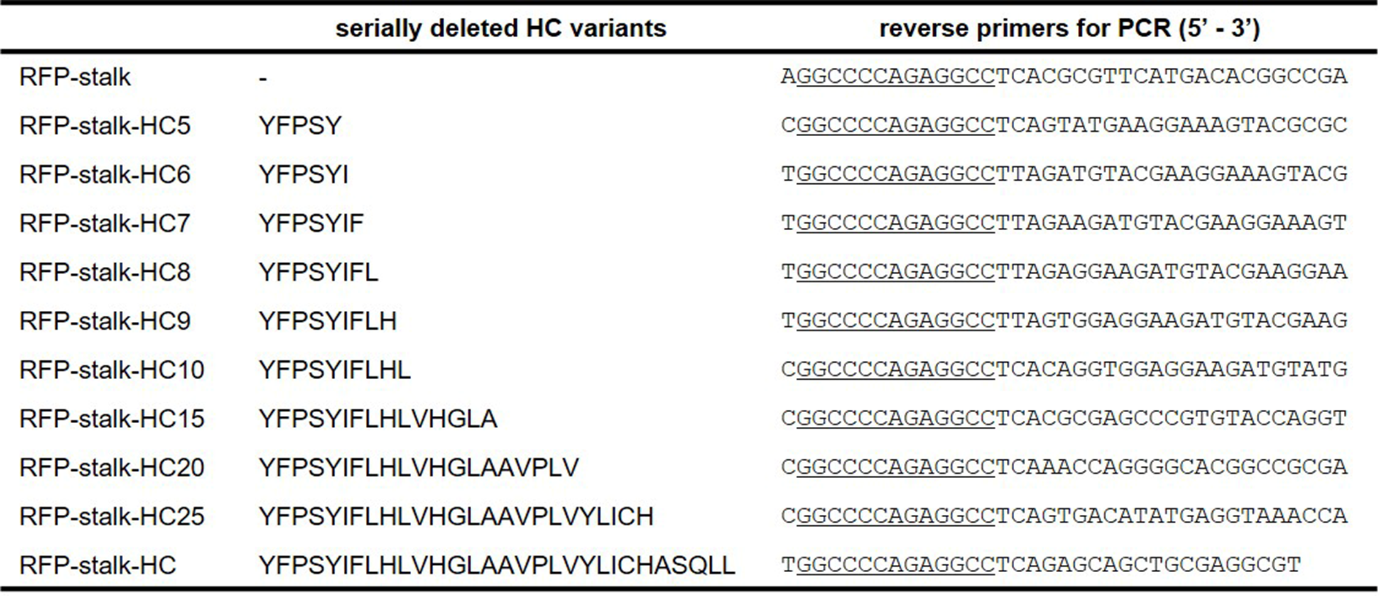


**SUPPLEMENTARY FIGURE LEGENDS**

**
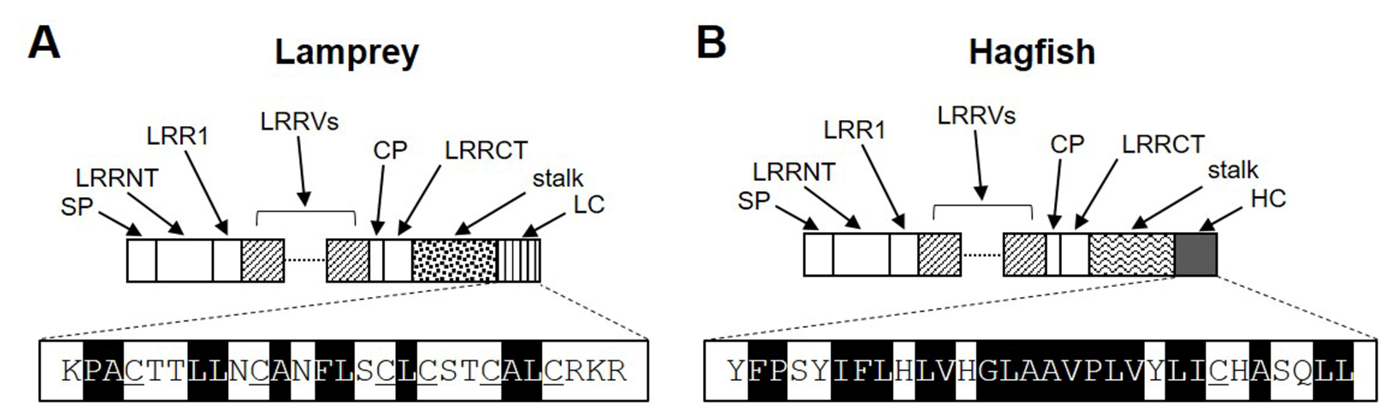
**

**Supplemental figure S1. Comparison of the structural domains of VLRBs in lampreys (A) and hagfish (B)**. The initial signal peptide (SP) is followed in sequence by: multiple leucine-rich repeat (LRR) domains encoding N-terminal capped LRR (LRRNT); variable LRR modules including LRRVe (LRRVs, 1 to 8 modules in lampreys and from 1 to 9 modules in hagfish); the connecting peptide (CP); the C-terminal capped LRR (LRRCT); the stalk region, and the C-terminal tail. The lamprey C-terminus (LC, 27 amino acids) possesses a Cys-rich tail, whereas the hagfish C-terminus (HC, 30 amino acids) has a hydrophobic region. Underlined C, Cys; black box, hydrophobic amino acids.


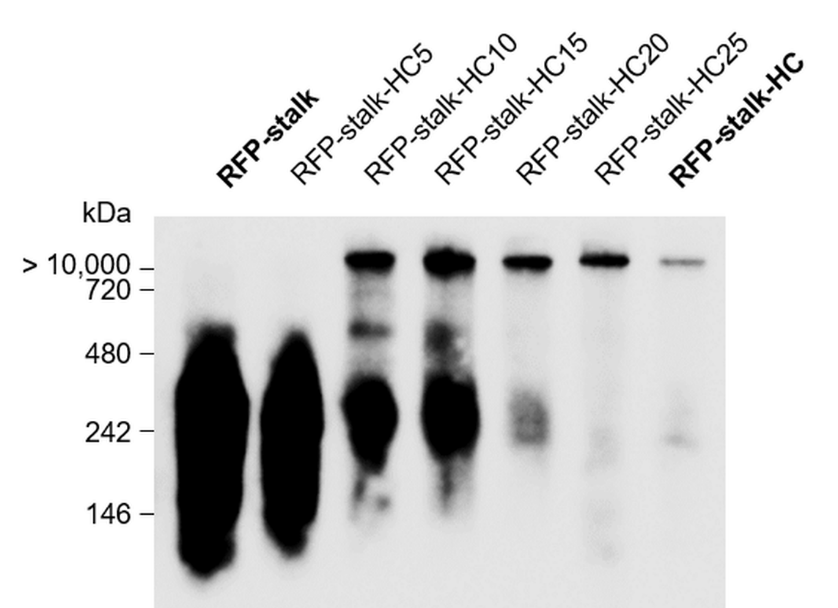


**Supplemental figure S2. Multimerization of RFP-stalk induced by the HC.** The secreted RFP-stalk with or without various versions of the HC (HC5, HC10, HC15, HC20, HC25 or HC) was separated by 8% native-PAGE under non-reducing condition, followed by Western blot analysis with 11G5.
